# Supplementary material for: Discovering adaptation-capable biological network structures using control-theoretic approaches
Source: PLoS Comput Biol. 2022 Jan 21;18(1):e1009769. doi: 10.1371/journal.pcbi.1009769 (PMC8809615; doi:10.1371/journal.pcbi.1009769)
Supplement: S1 Text — (PDF) [file pcbi.1009769.s001.pdf]

Supporting Information: Discovering adaptation-capable biological network structures using control-theoretic approaches

Priyan Bhattacharya[1,2,3], Karthik Raman[2,3,4*], Arun K. Tangirala[1,2,3*],

1 Department of Chemical Engineering, Indian Institute of Technology (IIT) Madras, Chennai, INDIA,
2 Robert Bosch Centre for Data Science and Artificial Intelligence (RBCDSAI), IIT Madras, Chennai, INDIA,
3 Initiative for Biological Systems Engineering (IBSE), IIT Madras, Chennai, INDIA,
4 Department of Biotechnology, Bhupat and Jyoti Mehta School of Biosciences, IIT Madras, Chennai, INDIA,

*kraman@iitm.ac.in(KR); *arunkt@iitm.ac.in(AT)

This section presents the necessary calculations, proofs and the rate laws used for simulation studies.

1 Two-node networks

Considering the inability of a single protein network to provide adaptation, we now turn to a two-protein network. The network comprises two proteins $\mathcal{C}$ and $\mathcal{A}$, which are connected; $\mathcal{A}$ is further connected to the external source of disturbance (input $\mathcal{D}$), and the concentration of $\mathcal{C}$ is considered as the "output species". Let us denote the concentration of $\mathcal{A}$, $\mathcal{C}$ and the disturbance species $\mathcal{D}$ by $x_1(t)$, $x_2(t)$,and $d(t)$ respectively. The resultant linearized state space representation is:

$$\dot{\mathbf{x}} = \mathbf{A}\mathbf{x} + \mathbf{B}d \tag{1}$$

$$\dot{\mathbf{x}} = \begin{bmatrix} a_{11} & a_{12} \\ a_{21} & a_{22} \end{bmatrix} \begin{bmatrix} x_1 \\ x_2 \end{bmatrix} + \begin{bmatrix} \alpha_1 \\ 0 \end{bmatrix} d \tag{2}$$

According to the previously derived conditions for adaptation (Eq.5 and Eq. 9 of the paper), the output state x_2 has to be controllable by the applied input. This demands a non-zero value for a_{21}, *i. e.* there should exist an edge from $\mathcal{A}$ to $\mathcal{C}$. As per the second condition for adaptation, the final value of the linearized output state x_2 should be zero, and the system matrix **A** should be Hurwitz.
Denote the steady-state value as $\mathbf{x}^* = \begin{bmatrix} x_1^* & x_2^* \end{bmatrix}^T$. Then, at steady state,

$$\begin{bmatrix} 0 \\ 0 \end{bmatrix} = \begin{bmatrix} a_{11} & a_{12} \\ a_{21} & a_{22} \end{bmatrix} \begin{bmatrix} x_1^* \\ x_2^* \end{bmatrix} + \begin{bmatrix} \alpha_1 \\ 0 \end{bmatrix} d \tag{3}$$

For any vector of the form $\begin{bmatrix} x_1^* & 0 \end{bmatrix}^T$ to be a solution to the above system of equations requires a_{21} to be zero. This is a violation of the controllability condition. Therefore, it can be concluded that a two-node network with different input–output nodes *cannot provide adaptation.*
To examine an alternate possibility, let us now consider the input node $\mathcal{A}$ itself as the output node as well. Note that the state x_1 is always controllable by the disturbance $\forall \alpha_1 \neq 0$. Also, if a_{22} is made zero possibly with a positive self loop on $\mathcal{C}$, then, the final steady-state value of x_1 can be zero, irrespective of x_2. In this case, for **A** to be a stable, $a_{21}a_{12}$ has

to be negative. This condition maps to a negative feedback between $\mathcal{A}$ and $\mathcal{C}$ (as shown in Fig 3 of the main text). Taken together, the admissible topology must have

1. $a_{22} = 0, \implies$ possible positive self loop on $\mathcal{C}$
2. $a_{21}a_{12} < 0 \implies$ negative feedback between $\mathcal{A}$ and $\mathcal{C}$.

1.1 Toilet Flush Phenomenon

To demonstrate further, let us consider a network of three proteins, $\mathcal{X}_1$, $\mathcal{X}_2$, and $\mathcal{X}_3$, where $\mathcal{X}_1$ is connected with $\mathcal{X}_2$, $\mathcal{X}_2$ is connected with $\mathcal{X}_3$, and $\mathcal{X}_3$ is connected with $\mathcal{X}_1$. Let the output node, $\mathcal{X}_1$, be perturbed with an input, u. If we adopt mass-action kinetics and assume the total mass to be conserved, *i. e.* $[\mathcal{X}_1] + [\mathcal{X}_2] + [\mathcal{X}_3] = 1$, thereby leaving two independent states, the state equation can be written as

$$[\dot{\mathcal{X}_1}] = k_1 u(1 - [\mathcal{X}_1] - [\mathcal{X}_2]) - k_2[\mathcal{X}_1]$$
$$[\dot{\mathcal{X}_2}] = k_2[\mathcal{X}_1] - k_3[\mathcal{X}_2]$$

For the case of zero input, the steady-state values are $[\mathcal{X}_1]^* = [\mathcal{X}_2]^* = 0$. It can be shown by our method that, for adaptation, k_3 has to be zero, but after a single step, the steady-state values of the states become [0, 1], thereby rendering the system linearized around the new steady-state uncontrollable. Hence the voltage gated Na^+ channel responds only to the first step change in the environment.

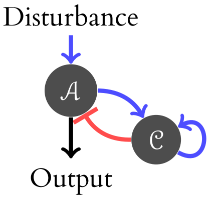

Fig S1. Admissible two-node topologies. The normal (blue) arrowheads signify activation, while the bar-headed (red) arrows signify repression.

2 Equivalence between conditions between adaptation

It was shown in the previous literature that the condition for adaptation is 1) one of the zeros in the transfer function to be placed in the origin. 2) In this work, we have shown for a system $(\mathbf{A}, \mathbf{B}, \mathbf{C}, \mathbf{D})$ to provide adaptation the necessary condition is $\mathbf{C}\mathbf{A}^{-1}\mathbf{B} = 0$. We argue that these two claims are equivalent. To prove this claim, we first establish $1 \rightarrow 2$.
Proof A proper and stable transfer function $H(s)$ which provides adaptation can be expressed as

$$H(s) = \frac{N_{n-1}s^{n-1} + N_{n-2}s^{n-2} + \cdots + N_1 s}{\alpha_{n-1}s^n + \alpha_{n-1}s^{n-1} + \cdots + \alpha_1 s + \alpha_0} \tag{4}$$

The corresponding state space representation $(\mathbf{A}, \mathbf{B}, \mathbf{C}, \mathbf{D})$ can be written assuming zero pole zero cancellation (full controllbility) can be obtained as

$$\mathbf{A} = \begin{bmatrix} 0 & 1 & \cdots & 0 \\ 0 & 0 & 1\cdots & 0 \\ \vdots & \vdots & \vdots & \vdots \\ -\alpha_0 & -\alpha_1 & -\alpha_2\cdots & -\alpha_{n-1} \end{bmatrix}, \mathbf{C} = \begin{bmatrix} 0 & N_1 & N_2\cdots & N_{n-1} \end{bmatrix}, \mathbf{B} = \begin{bmatrix} 0 \\ 0 \\ \vdots \\ 1 \end{bmatrix}, \text{and } \mathbf{D} = 0$$

With the structure of $(\mathbf{A}, \mathbf{B}, \mathbf{C}, \mathbf{D})$ it can be seen that $\mathbf{CA}^{-1}\mathbf{B} = 0$ which proves the forward assertion.
Subsequently, it is to be proved that the zero at origo condition amounts to the condition derived in the main script.
Proof: For a given state space structure $(\mathbf{A}, \mathbf{B}, \mathbf{C}, \mathbf{D})$ the transfer function can be written as

$$H(s) = \mathbf{C}(s\mathbf{I} - \mathbf{A})^{-1}\mathbf{B}$$

. The zero at the origo means zero final value of the step response $(Y(s))$ of the system.

$$Y(s) = \frac{H(s)}{s} \tag{5}$$

$$\lim_{t\to\infty} y(t) = \lim_{s\to 0} sY(s) \tag{6}$$

$$\lim_{t\to\infty} y(t) = \lim_{s\to 0} H(s)) \tag{7}$$

$$\lim_{t\to\infty} y(t) = \lim_{s\to 0}(\mathbf{C}(s\mathbf{I} - \mathbf{A})^{-1}\mathbf{B}) \tag{8}$$

$$\implies \mathbf{C}(\mathbf{A})^{-1}\mathbf{B} = 0 \tag{9}$$

So, it can be seen that both the assertion and its converse are true so the condition for adaptation derived in this work is equivalent to the standard condition of zero at the origo.

2.1 Derivation of Eq 34 from Eq 9 of the paper

: In this subsection, we argue that the infinite precision condition derived in equation Eq 9 is a more general than the one derived in Eq 34. The infinite precision condition is obtained as

$$\mathbf{CA}^{-1}\mathbf{B} = 0 \tag{10}$$

For the specific case of Eq 34, the input disturbance is applied on the first node and the output is considered as the concentration of the kth node. Therefore the $\mathbf{B} \in \mathbb{R}^N$ and $\mathbf{C} \in \mathbb{R}^{1\times N}$ matrix are of the form $\beta\mathbf{e}_1$ and $\zeta\mathbf{e}_k^T$ respectively where $\mathbf{e}_j \in \mathbb{R}^N$ are unit vectors across the jth axis and β, ζ are nonzero scalars. It is to be noted that since $\mathbf{A}$ is Hurwitz as per the stability condition the determinant is non-zero and will be the denominator in the expression of $\mathbf{CA}^{-1}\mathbf{B}$. According to the very definition of matrix inverse we know that the $(i,j)^{th}$ element of $\det(\mathbf{A})\mathbf{A}^{-1}$ refers to the minor of the $(j,i)^{th}$ component of $\mathbf{A}$. Due to the specific structure of $\mathbf{B}$, $\mathbf{A}^{-1}\mathbf{B}$ will be a scaled version of the first column of $\mathbf{A}^{-1}$. Similarly with the given structure of $\mathbf{C}$ the expression $\mathbf{CA}^{-1}\mathbf{B}$ returns a scaled version of the $(k,1)^{th}$ element of $\det(\mathbf{A})\mathbf{A}^{-1}$ which in turn is the minor of the $(1,k)^{th}$ component of the $\mathbf{A}$ matrix.

3 Generalization

This section deals with the necessary results and demonstrations that act as the stepping stones for the results shown in the main text.

Theorem 1. *An N-node controllable network with multiple loops and no common nodes cannot provide adaptation if the effective signs of all the loops are positive.*

Proof. Let $\mathbb{Q}$ be the set of all the controllable candidate motifs containing multiple loops with no common nodes but edges connecting each loop. Further, it is evident from the statement of the theorem that every node is involved in exactly one loop. Suppose, an element $\mathcal{P}$ in $\mathbb{Q}$ consists of L_p number of loops. It is evident that for $\mathcal{P}$ to be controllable, it has to contain at least N edges. Therefore, the controllability condition requires $\mathcal{P}$ to have $N + L_p - 1$ edges. In this present context, the minimal motifs can be thought of as the elements in $\mathbb{Q}$ which has $N + L_p - 1$ number of edges and L_p number of loops. Define the set $\Phi \subset \mathbb{Q}$ consisting of all possible minimal motifs in $\mathbb{Q}$.
In order for a minimal motif in Φ to provide adaptation, it must satisfy the adaptation condition Eq 34. This can be achieved if and only if at least one of the diagonal elements of the $\mathbf{A}$ matrix is zero (refer to *SI*). Let us assume, that the D_z^{th} row of the $\mathbf{A}$ matrix contains the zero diagonal. Since none of the loops share any common node, the D_z^{th} node must be associated with only one loop

denoted by L_z. Suppose L_z involves N_z number of nodes. The set Ω contains all the elements in $\mathbf{A}$ matrix that correspond the loop L_z. Suppose $\mathbf{A}_z \in \mathbb{R}^{N_z \times N_z}$ be the sub matrix of $\mathbf{A}$ that captures the connection patterns of all the N_z nodes involved in L_z. Since each node is involved in only one loop the structure of the associated $\mathbf{A}$ matrix can be written as

$$\mathbf{A} = \begin{bmatrix} \mathbf{A}_u & \mathbf{0} & \mathbf{0} \\ \mathbf{Q}_1 & \mathbf{A}_Z & \mathbf{0} \\ \mathbf{Q}_2 & \mathbf{Q}_3 & \mathbf{A}_{N_z} \end{bmatrix} \tag{11}$$

where, $\mathbf{A}_u \in \mathbb{R}^{N_u \times N_u}$ is the sub matrix that captures the upstream loops to L_z and $\mathbf{A}_{N_z} \in \mathbb{R}^{(N-N_z)\times(N-N_z)}$ involves all the loops except the upstream loops and L_z. The sub matrix $\mathbf{Q}_i$ captures the downward edges joining the loops. If the spectrum of $\mathbf{A}_z$, and $\mathbf{A}_{N_z}$ are $\nu_{\mathbf{A}_z}$ and $\nu_{\mathbf{A}_{N_z}}$ respectively then the spectrum of $\mathbf{A}$ ($\nu_{\mathbf{A}}$) can be expressed as

$$\nu_{\mathbf{A}} = \nu_{\mathbf{A}_u} \bigcup \nu_{\mathbf{A}_z} \bigcup \nu_{\mathbf{A}_{N_z}} \tag{12}$$

It is evident from equation (12) that for $\mathbf{A}$ to be Hurwitz, $\mathbf{A}_z$ has to be Hurwitz. Imposing the stability criterion as defined in the equation Eq 26 on $\mathbf{A}_z$,

$$sign(|\mathbf{A}_z|) = (-1)^N_z \tag{13}$$

Since one of the diagonal components of $\mathbf{A}_z$ is zero, the determinant in this case is the product of all the elements mapping to all the edges involved in the L_z. From combinatorial matrix theory [1], the sign assigned to a loop with N_z number of nodes in the determinant of a matrix can be written as $(-1)^{(N_z)-1}$.

$$|\mathbf{A}_z| = (-1)^{(N_z-1)} \prod_{i=1}^{N_z} \alpha_i, \ \alpha_i \in \Omega \tag{14}$$

Therefore, using equation (13) we can say for $\mathbf{A}$ to be Hurwitz the following condition should hold

$$sign(|\mathbf{A}_z|) = sign((-1)^{(N_z-1)}) sign\left(\prod_{i=1}^{N_z} \alpha_i\right) \tag{15}$$

$$(-1)^N_z = sign((-1)^{(N_z-1)}) sign\left(\prod_{i=1}^{N_z} \alpha_i\right) \tag{16}$$

$$\implies sign\left(\prod_{i=1}^{N_z} \alpha_i\right) = -1 \tag{17}$$

From (17), it is clear that, if the cumulative signs for all the loops of any candidate motif in Φ are positive then the resultant $\mathbf{A}$ becomes unstable, failing to provide adaptation. □

Theorem 2. *An* $N-$ *node network containing multiple loops with no common species and no edge connecting the loops cannot provide adaptation.*

Proof. As established in the methodology section, the underlying linearized dynamical system has to be controllable by the external disturbance input to perform adaptation. Now, for an N-edge network with L_m loops with no common nodes between them, the associated system matrix $\mathbf{A}$ can be written as

$$\mathbf{A} = \begin{bmatrix} \mathbf{S} & \mathbf{0} \\ \mathbf{0} & \mathbf{T} \end{bmatrix} \tag{18}$$

where, $\mathbf{s}$ consists of the loop element involving the input node x_1 and $\mathbf{T}$ comprises elements representing all the remaining $L_m - 1$ loops. To avoid the trivial scenario, we consider that the output node is not involved in any loop with the input network. Let the loop involving the input node x_1 involves n_1 number of nodes then $\mathbf{S} \in \mathbb{R}^{N_1 \times N_1}$ and $\mathbf{T} \in \mathbb{R}^{(N-N_1)\times(N-N_1)}$. It is to be noted that if the output node is involved in a loop with the input node, then the effective network order reduces to N_1. In order to avoid such trivial cases, we assume that the output is involved in any of

the remaining $L_m - 1$ loops. Given $\mathbf{B} = \begin{bmatrix}\beta & 0...\end{bmatrix}^T = \begin{bmatrix}\mathbf{B}_{s1\times n_1} & \mathbf{0}_{1\times(n-n_1)}\end{bmatrix}^T$ associated Kalman controllability matrix ($\mathbf{K}$) for the pair ($\mathbf{A}, \mathbf{B}$) can be evaluated as

$$\mathbf{K} = \begin{bmatrix}\mathbf{B}_s{}^T & \mathbf{S}\mathbf{B}_s{}^T & \mathbf{S}^2\mathbf{B}_s{}^T & \cdots & \mathbf{S}^{(N-1)}\mathbf{B}_s{}^T \\ \mathbf{0} & \mathbf{0} & \mathbf{0} & \cdots & \mathbf{0}\end{bmatrix} \tag{19}$$

$$\implies \dim(\mathrm{Im}(\mathbf{K})) \leqslant \dim(\mathrm{Im}(\mathbf{S})) < N \tag{20}$$

where, $\mathrm{Im}(\cdot)$ denotes the column space of a matrix, and $\dim(\cdot)$ calculates the dimension of a given vector space. From (20), it is clear that the Kalman rank condition is not satisfied in this case, leading to failure in achieving adaptation. □

Therefore, the problem of uncontrollability discussed in Theorem 2 can be circumvented by placing at least one connecting edge between each loop with no common nodes. In that case, the question of stability has to be taken into consideration.

3.1 Two principal means of achieving infinite precision

: The infinite precision equation represented in Eq 34 involves computation of the minor of the term that maps back to an edge from the output to the input node. In an $N-$node network ($x_1, X-2, \cdots, x_N$ as the concentration of the 1st, 2nd, $\cdots$, N^{th} node respectively), if the concentration of the input node is considered as the first node (concentration x_1) and the kth node as output with the respective concentration expressed as x_k, then according to Eq 34, the

$$\tilde{\mathbf{A}} := \mathrm{minor}(\mathbf{A}_{1k}) = \mathrm{minor}\left(\frac{\partial \dot{x}_1}{\partial x_k}\right)\Bigg|_{x^*} = 0$$

. For the system matrix $\mathbf{A} \in \mathbb{R}^{N\times N}$ there are $N!$ number of terms present in the determinant expression in which $\mathbf{A}_{1k}\tilde{\mathbf{A}}$ involves $(N-1)!$ number of terms. From combinatorial matrix theory, it is well known that ([1]) each term in the determinant expression of any diagraph matrix can be expressed as the product of the diagonal entries and loops with no mutual nodes. Following this, it can be said that each term of $\tilde{\mathbf{A}}$ contains exactly one forward path from the input to the output node. It is to be noted that each of the $(N-1)!$ terms in the expression of $\tilde{\mathbf{A}}$ contains $N-1$ elements. In terms which refer to the forward paths with less than $N-1$ number of edges, the remaining entries are composed of the diagonal and the loop elements. It is obvious that there are two ways in which all the terms of $\tilde{\mathbf{A}}$ sum up to zero

1. All the terms are zero individually.
2. There exist terms with equal and opposing actions.

As discussed earlier $\tilde{\mathbf{A}}$ contains $(N-1)!$ terms. Each term contains exactly one forward path from the input to the output node. One option can be to have a network without any forward path but this leads to uncontrollability of the output node. So the only other option is to make all the forward paths with $N-1$ edges absent along with at least one of of the diagonal elements to be zero such that all the terms are individually zero. This is exactly what is referred as the opposer module in [2]
In the second case, the non-zero terms can be grouped in to three classes. In this context, let us define certain notations and functions that shall be helpful in putting things in perspectives. Suppose set $\mathbb{N}_{PL}$ contains all the forward paths and loops of the network, set $\mathbb{V}$ contains all the nodes. Also, $\mathbb{N} : \mathbb{N}_{PL} \to \mathbb{D} \subset \mathbb{V}$ returns all the nodes involved in a given forward path $\mathcal{P} \in \mathbb{N}$. Then, cardinality of the set $\mathbb{N}(\mathcal{P})$ provides the number of nodes involved in the forward path $\mathcal{P}$. $\mathbf{A}_{P\in\mathbb{S}}$ refers to the component of the $\mathbf{A}$ matrix that represents P. Since, $\mathbf{A}$ acts as the diagraph matrix this is a ono-to-one mapping. Therefore, P and $\mathbf{A}_P$ shall be used interchangeably to reduce the abundant notations.
i) Let us consider two forward paths $F_1 \in \mathbb{N}_{PL}$ with f_1 nodes, $F_2 \in \mathbb{N}_{PL}$ with f_2 nodes and a loop L involving p nodes such that $\mathbb{N}(L \cap (F_1 \cup F_2)) = \Phi$. Σ_{f1} is the permutation set of all diagonals $(N - f_1 - p - 1)$ except the ones situated in $\mathbb{N}(L) \cup \mathbb{N}(F_1)$ similar notations are also invoked for F_2. For

this case, the expression of $\tilde{\mathbf{A}}$ concerning the aforementioned loops. forward paths can be written as

$$
\begin{aligned}
\tilde{\mathbf{A}} &= (-1)^{f_1+p-1}\sum_{\Sigma_{f1}} F_1 L D_{\sigma_{N+f_1-p-1}} \\
&+ (-1)^{f_2+p-1}\sum_{\Sigma_{f2}} F_2 L D_{\sigma_{N+f_2-p-1}} \\
&+ (-1)^{f_1-1}\sum_{\Sigma_{f1D}} F_1 L D_{\sigma_{N-f_1-1}} \\
&+ (-1)^{f_2-1}\sum_{\Sigma_{f2D}} F_1 L D_{\sigma_{N+f_2-p-1}} \\
\implies & (-1)^{N-2} L\Big(\sum_{\Sigma_{f1}} F_1 |D_{\sigma_{N+f_1-p-1}}| + \sum_{\Sigma_{f2}} F_2 |D_{\sigma_{N+f_2-p-1}}|\Big) \\
&+ (-1)^{N-1}\Big(\sum_{\Sigma_{f1D}} F_1 |D_{\sigma_{N-f_1-1}}| + \sum_{\Sigma_{f2D}} F_1 |D_{\sigma_{N+f_2-p-1}}|\Big)
\end{aligned}
$$

Now, the only way to achieve $\tilde{\mathbf{A}} = 0$ while ensuring stability ((12)) is to have $\mathtt{sign}(F_1) = (-1)\mathtt{sign}(F_2)$ ii) Let us consider two forward paths $F_1 \in \mathbb{N}_{PL}$ with f_1 nodes, $F_2 \in \mathbb{N}_{PL}$ with f_2 nodes and two loops L_1, L_2 involving p_1 and p_2 nodes such that $\mathbb{N}(L_1) \cap \mathbb{N}(F_2) = N_j$, $\mathbb{N}(L_2) \cap \mathbb{N}(F_1) = N_k$ and $\mathbb{N}(L_1) \cap \mathbb{N}(L_2) = N_l$. It is to be noted that in this case, apart from F_1 and F_2 there exist two other forward paths 1) From the input node (denote as node 1) to the N_k^{th} node via F_1, then from N_k^{th} to the N_l^{th} node via L_2 and lastly from N_l^{th} to the N_j^{th} via L_1 and from N_j^{th} to output node (denote as k^{th} node) via F_2. Let us call this as F_{12} 2) From the input node (denote as node 1) to the N_j^{th} node via F_2, then from N_j^{th} to the N_l^{th} node via L_1 and lastly from N_l^{th} to the N_k^{th} via L_2 and from N_k^{th} to output node via F_1. Let us denote this as F_{21} In this case as well the terms in the expression of $\tilde{\mathbf{A}}$ shall be similar to the previous case except an addition of two forward paths F_1 and F_2. Now, the only way to mutually cancel the terms in $\tilde{\mathbf{A}}$ concerning the forward path F_1 and F_2, assuming F_1 and F_2 are of the same sign is to have $\mathtt{sgn}(L_1) = (-1)\mathtt{sgn}(L_2)$ in that case it can be seen that $\mathtt{sgn}(F_{12}F_{21}) = \mathtt{sgn}(L_1L_2) = -1$. This means the forward paths F_{12} and F_{21} are of the opposite sign. iii) Let us consider two forward paths $F_1 \in \mathbb{N}_{PL}$ with f_1 nodes, $F_2 \in \mathbb{N}_{PL}$ with f_2 nodes and two loops L_1, L_2 involving p_1 and p_2 nodes such that $\mathbb{N}(L_1) \cap \mathbb{N}(F_2) = N_j$, $\mathbb{N}(L_2) \cap \mathbb{N}(F_1) = N_k$ and $\mathbb{N}(L_1) \cap \mathbb{N}(L_2) = \Phi$. The corresponding expression for $\tilde{\mathbf{A}}$ can be written as

$$
\begin{aligned}
\tilde{\mathbf{A}} &= (-1)^{f_1+p_1-1} F_1 L D_{\sigma_{N-f_1-p_1-1}} \\
&+ (-1)^{f_1-1} F_1 D_{\sigma_{N-f_1-1}} \\
&+ (-1)^{f_2+p_2-1} F_2 L D_{\sigma_{N-f_2-p_2-1}} \\
&+ (-1)^{f_2-1} F_1 D_{\sigma_{N+f_2-1}} \\
\implies & (-1)^{f_1} F_1 D_{\sigma_{N-f_1-p_1-1}} \underbrace{\Big((-1)^{p_1-1} L_1 + F_1 D_{\sigma_{p_1}}\Big)}_{\mathbb{D}_{L_1}} \\
&+ (-1)^{f_2} F_2 D_{\sigma_{N-f_2-p_2-1}} \underbrace{\Big((-1)^{p_2-1} + F_1 D_{\sigma_{p_2}}\Big)}_{\mathbb{D}_{L_1}} \\
\implies & (-1)^{p_1} F_1 |D_{\sigma_{N-f_1-p_1-1}}| \mathbb{D}_{L_1} \\
&+ (-1)^{p_2} F_2 |D_{\sigma_{N-f_2-p_2-1}}| \mathbb{D}_{L_1}
\end{aligned}
$$

Assume F_1 and F_2 are of the same sign then

$$\implies \quad (-1)^{p_1}\mathbb{D}_{L_1} + (-1)^{p_2}\mathbb{D}_{L_1} = 0 \tag{21}$$

Again, for stability, we know

$$sgn\left(D_{N-p_1-p_2}\mathbb{D}_{L_1}\mathbb{D}_{L_2}\right) = (-1)^N \tag{22}$$

$$sgn\left(\mathbb{D}_{L_1}\mathbb{D}_{L_2}\right) = (-1)^{p_1+p_2} \tag{23}$$

The only way to satisfy (21) $sign\left(\mathbb{D}_{L_1}\right) = (-1)^{p_1+2m+1}$, $sign\left(\mathbb{D}_{L_2}\right) = (-1)^{p_2+2m}$ or $sign\left(\mathbb{D}_{L_1}\right) = (-1)^{p_1+2m}$, $sign\left(\mathbb{D}_{L_2}\right) = (-1)^{p_2+2m+1}$ where, $m \in \mathbb{I}^+$. In both the cases $sign\left(\mathbb{D}_{L_2}\mathbb{D}_{L_1}\right) = (-1)^{p_1+p_2+2m+1} \implies (-1)^{p_1+p_2+1}$

This again is the violation of the stability condition depicted in (23). Therefore the only way to drive $\tilde{\mathbf{A}}$ to zero is to have incoherrent feedforward paths considering all the diagonal elements are non-zero and negative.

It has already been established in the main text that in order for the network to be able to provide adaptation, it has to be controllable with respect to the external disturbance. In the following theorem, we argue that there exists at least one forward path from the input to the output node for the system to be controllable.

Theorem 3. *For an $N-$node network with different input and output nodes, considering the states as the concentration of the proteins the resultant state space system is output controllable if there exists at least one forward path from the input to the output node.*

Proof. In order to prove the above theorem, we have to show that the system is not output controllable if there exists no forward path from the input node to the output node.

Without any loss of generality, let us denote the input node as the first node with concentration x_1 and the same for the kth node (x_k) is considered as the output. Assume, there are p nodes which are connected with the input node in such a way that there exists at least one forward path from the input node to all of the P nodes. None of the remaining $N-P$ nodes can be reached from the input node. Using the property that the system matrix $\mathbf{A}$ for the linearised state space system acts as a digraph matrix for the network,

$$\mathbf{A} = \begin{bmatrix} \mathbf{A}_1 & \mathbf{A}_{12} \\ \mathbf{A}_{21} & \mathbf{A}_2 \end{bmatrix} \tag{24}$$

where, $\mathbf{A}_1 \in \mathbb{R}^{P\times P}$ captures the inter connections among the P nodes reachable from the input node, $\mathbf{A}_{12} \in \mathbb{R}^{P\times N-P}$ contains the connections from the $N-P$ nodes to the first P nodes, $\mathbf{A}_{12} \in \mathbb{R}^{N-p\times P}$ contains the connections from the first P nodes to the remaining $N-P$ nodes, and $\mathbf{A}_2 \in \mathbb{R}^{N-P\times N-P}$ reflects the interconnections among the last $N-P$ nodes. Since there exists no froward path from the input node to any of the $N-P$ nodes $\mathbf{A}_{21}$ is a zero matrix. The actuator matrix $\mathbf{B}$ can be written as

$$\mathbf{B} = \begin{bmatrix} \beta & 0 & \cdots & 0 \end{bmatrix}^T = \begin{bmatrix} \mathbf{B}_1 & \mathbf{0} \end{bmatrix}^T \tag{25}$$

where, $\mathbf{B}_1 \in \mathbb{R}^{1\times P}$ is an elementary vector with the first element being non-zero (β) as the input node is considered as the first node. Given the pair $(\mathbf{A}, \mathbf{B})$ the controllability matrix (Γ_c) can be written as

$$\mathbf{A} = \begin{bmatrix} \mathbf{A}_1 & \mathbf{A}_{12} \\ \mathbf{A}_{21} & \mathbf{A}_2 \end{bmatrix}, \ \mathbf{B} = \begin{bmatrix} \mathbf{B}_1 & \mathbf{0} \end{bmatrix}^T \tag{26}$$

$$\Gamma_c = \begin{bmatrix} \mathbf{B}_1^T & \mathbf{A}_1\mathbf{B}_1^T & \cdots & \mathbf{A}_1^{N-1}\mathbf{B}_1^T \\ \mathbf{0} & \mathbf{0} & \cdots & \mathbf{0} \end{bmatrix} \tag{27}$$

$$\implies \dim(Im(\Gamma_c)) \leqslant \dim(Im(\mathbf{A}_1)) < N \tag{28}$$

where $Im(.)$ denotes the column space of a matrix and $\dim(.)$ calculates the dimension of a given vector space. From (28) it is clear that the Kalman rank condition can not be achieved in this case. □

3.2 Necessary results for Theorem 5 of the paper

In this section, we shall delve into the necessary details that are instrumental to the proof of theorem Theorem 5 in the main manuscript. We first present a constructive method to demonstrate the number of exchanges of indices required for any loop with k nodes is $k-1$ thereby making the associated prefix sign to be $(-1)^{k-1}$. Subsequently, we derive a number of important results that enables the application of mathematical induction technique adopted for the proof of theorem.

3.2.1 Number of exchanges required for a $k-$node loop

Let us assume a loop $\mathcal{L}_k$ engaging nodes $x_{t_j}, \forall j = 1(i)k$ such that, the term in the characteristic polynomial of the associated digraph matrix $\mathbf{A}$ that represents $\mathcal{L}_k$ can be written as

$$\mathbf{A}(\mathcal{L}_k) = S_k \times \mathbf{A}_{t_1,t_2}\mathbf{A}_{t_2,t_3}\mathbf{A}_{t_3,t_4}\mathbf{A}_{t_4,t_5}\cdots\mathbf{A}_{t_{k-1},t_k}\mathbf{A}_{t_k,t_1} \tag{29}$$

where, S_k is the prefix sign which is determined by the number of required manipulations to covert the loop element to product of k diagonals. Since $\mathcal{L}_k$ contains k nodes $\mathbf{A}(\mathcal{L}_k)$ is composed of k elements- each from the columns (rows) in the set $\{t_i\}_1^k$.

From (29), the first obvious exchange can be between the column index of $\mathbf{A}_{t_1,t_2}$ with the same of $\mathbf{A}_{t_2,t_3}$ leaving the modified first term as $\mathbf{A}_{t_1,t_3}$ with a subsequent exchange of the row element of the first term with $\mathbf{A}_{t_3,t_4}$. Repeating this exercise for $k-2$ times we get

$$\tilde{\mathbf{A}}(\mathcal{L}_k) = S_k \times \prod_{j=2}^{k-1}\mathbf{A}_{t_1,t_k}\mathbf{A}_{t_j,t_j}\mathbf{A}_{t_k,t_1}$$

Therefore, the final exchange between the column index of $\mathbf{A}_{t_1,t_k}$ and the same for $\mathbf{A}_{t_k,t_1}$ results in product of k diagonals rendering the total number of exchanges to $k-1$. Therefore the prefix sign S_k can be determined as $(-1)^{k-1}$.

3.2.2 Necessary results for $N-$node network with no negative feedbacks

The coefficient E_k of s^{N-k} in the characteristic polynomial of $\mathbf{A}$ can be expressed as

$$E_k = \sum_{p=2}^{k}\sum \mathcal{L}_{i,j}^{p}\left((-1)^{k+p-1}\mathbb{S}^{(k-p)}_{\mathcal{L}^p_{i,j}}\right) + R_k + \sum_{i=1}^{{}^N C_k}(-1)^k\sigma_k^i \mathrm{Diag}(\mathbf{A}) \quad \left(\mathbb{S}^{(k-p)}_{\mathcal{L}^p_{i,j}} := \sum\left[\mathbb{S}^{k-p}\Big/\mathbb{D}_{\mathcal{L}_j^{k-p}}\right]\right) \tag{30}$$

As described in the main text, that each element of the first two terms in (30) contains at least one loop. In the scenario, $k = 1(i)3$, each element shall contain exactly one loop. Therefore from Eq 45 of the main text, all the elements are negative for $k = 1(i)3$.

For $k \geqslant 4$, the elements of the first two terms in the expression of E_k may contain multiple loop. We shall first investigate the case of the first term. Let us assume that the element containing i^{th} term in the set $\mathcal{L}_j^p$ is positive for any $k \geqslant 4$ and greater in absolute value than all the other elements with negative sign. Since, all the loops are assumed to be of positive sign the sign of $(-1)^{k+p-1}\mathbb{S}^{(k-p)}_{\mathcal{L}^p_{j,i}}$- the term associated with the $\mathcal{L}^p_{j,i}$ in (30) has to be positive. It can be easily verified that if all the elements in $(-1)^{k+p-1}\mathbb{S}^{(k-p)}_{\mathcal{L}^p_{j,i}}$ are positive then the corresponding expression for E_{k-p+1} shall be negative. Let us assume there exist a set of $\mathbb{N}_l$ of cardinality l containing elements in $(-1)^{k+p-1}\mathbb{S}^{(k-p)}_{\mathcal{L}^p_{j,i}}$ that are positive and greater than the rest of negative components in absolute terms. Further, the expression E_{k-p+1} can also be written as the second and the third term of E_{k-p+1} can be combined as

$$R_{k-p+1} + \sum_{i=1}^{{}^N C_{k-p+1}}(-1)^{k-p+1}\sigma^i_{k-p+1}\mathrm{Diag}(\mathbf{A}) = \sum\left((-1)^{k-p+1}\mathbb{S}^{(k-p)}_{\mathcal{L}^p_{Q_p,j,l}}\alpha_l\right) \tag{31}$$

where, α_l is the sum of the diagonal elements that have no common indices with $\mathbb{S}^{(k-p)}_{\mathcal{L}^p_{Q_p,j,l}}$. Therefore, if we start from the element $\max\left(\mathbb{S}^{(k-p)}_{\mathcal{L}^p_{Q_p,j,l}}\right)$ accommodate all the positive elements in decreasing order in (31) followed by the loops which has $p-1$ nodes common with all the items in $\mathbb{N}_l$. It can be seen that if the α_l for the lth element in $\mathbb{S}^{(k-p)}_{\mathcal{L}^p_{Q_p,j,l}}$ has L diagonals then α_l for the $(l+1)^{th}$ element which has $p-1$ common rows and comlumns with the previous entry contains sum of $l-1$ recurring diagonals. Therefore, $|\alpha_{l+1}| < \alpha_l$. This implies the overall sign of E_{k-p+1} is negative (all the diagonal elements are negative).
The second term of (31) contains combination of the loops and their associated minors that do not contain the jth column (or row).For the second term of E_K to be positive, at least one element in R_k has to be positive. Without any loss of generality, let us assume that the ath term is positive and greater than the other negative terms. Furthermore, it has already been shown in the case for the first term of E_k that any positive principal minor with p rows and columns can not be greater than the sum of all the remaining principal minors with same number of rows and columns for then E_{p+1} shall be negative thereby failing to satisfy the stability condition in Eq 26. Therefore, the only option is

$$\left((\mathcal{L}^p/\mathcal{L}^p_j)_w - \underbrace{\prod \mathbf{A}_{i,i}}_{i \in \text{node}(\mathcal{L}^p/\mathcal{L}^w_j)_w} \right) > \sum_{i \neq w} (\mathcal{L}^p/\mathcal{L}^p_j)_i \left((-1)^{k+p-1} \mathbb{S}^{(k-p)}_{\mathcal{L}^p_{i,(\mathcal{L}^p/\mathcal{L}^p_j)_i}} \right) \tag{32}$$

Again, through the construction identical to the first case (as shown for the case of the first term of E_k), we can show that indeed if (32) is satisfied then E_{p+1} shall be negative rendering the system unstable. Therefore, the only amicable conclusion is the second term is also negative.

3.3 Hurwitz stability of Negative feedback loops and IFFLP

The Hurwitz condition in systems theory guarantees the exponential asymptotic stability of the linearised system. Further, the Hartman-Grobman theorem ensures the stability of the corresponding non-linear system if its linearised counter part is exponentially stable. Therefore, to comment on the stability of the actual non linear system, we first investigate whether the system matrix **A** of the linearised system is Hurwitz. For any matrix $\mathbf{A} \in \mathbb{R}^{N \times N}$ to be Hurwitz, one of the necessary conditions is the following

$$\sum_{k=1}^{N} M^i_{\mathbf{A}k} > 0 \forall i = 1(i)N \tag{33}$$

where, $M^i_{\mathbf{A}k}$ is the all possible i^{th} principal minors of **A**. It is evident from (33), there are N conditions that need to be satisfied for any $N \times N$ matrix to be Hurwitz. As established before, the linearised system matrix **A** can be considered as the diagraph matrix of the associated network structure. In this scenario, the sum of all possible i^{th} principal minors can be expressed as all possible $i-$node loops present in the network structure, loops with less than i nodes and diagonals. To illustrate further, assume the network has two loops L_1 and L_2 containing N_1 and N_2 number of nodes. Further, assume there exists no common nodes in L_1 and L_2. In that case, the expression for the sum of all i^{th} $(i > N_1 + N_2)$ principal minor can be written as

$$\sum_{k=1}^{N} M^i_{\mathbf{A}k} = (-1)^i \Big(\sum \sigma_{D_i} + (-1)^{N_1-1} \sigma_{D_{(i-N_1)|L_1}} \tag{34}$$

$$+ (-1)^{N_1-1} \sigma_{D_{(i-N_2)|L_2}} + (-1)^{N_1+N_2-2} \sigma_{D_{(i-N_1-N_2)|L_1,L_2}} \Big) \tag{35}$$

where, $\sigma_{i|t}$ is the permutation operator that chooses k diagonals from the set of N (**A** is $N \times N$) diagonal elements, the subscript t means the choice of i diagonal elements should be such that it does not have any common co-ordinate with the elements in t. For a network with a single loop

(L_p) of p_1 nodes and cumulative sign being negative the sum of all the principal minors of order i can be written as

$$\sum_{k=1}^{N} M_{Ak}^{i} = \begin{cases} (-1)^i\left(\sum \sigma_{D_i}\right) & i < p_1 \\ (-1)^i\left((-1)^{p_1-1}\sigma_{D_{(i-p_1)|L_p}} + \sum \sigma_{D_i}\right) & i \geqslant p_1 \end{cases}$$

It can be seen in both the scenarios ($i < p_1$, $i \geqslant p_1$) the sign of the sum of ith order minor is always positive given the diagonals and the L_1 is of negative sign. Hence presence of negative feedback loop satisfies the Hurwitz condition for exponential stability.
In the case of feedforward networks without any loop the sum of the ith principal minors shall always be sum of the combination of i diagonal elements chosen from N diagonals in which case, the sum of the principal minors shall always be positive $\forall i$ given the diagonal elements are negative. This also guarantees the Hurwitz property of the networks with only feedforward paths.
For an $N \times N$ matrix there are $N!$ number of terms present in the determinant expression. It can be proved that every term in the expression contains at least one loop except the product term of the diagonals. The elements which carried a single loop were discussed in the main text and it was shown that the elementary motif associated with one of these terms need to be of negative feedback type i,e the loop sign should be negative. The elements containing multiple non-overlapping loops can not provide adaptation for the associated network becomes uncontrollable. For these networks it can be shown that if the cumulative sign of all the loops are positive then also it can satisfy the determinant condition i,e, the sign of the determinant becomes $(-1)^N$. These networks along with another link/loop (to make the network controllable) leads to Hurwitz instability by making at least one of eigenvalues positive. Following is an illustration of a four node network. Assume a five node network which has two loops one involving $\mathcal{A}$, $\mathcal{B}$, $\mathcal{C}$ and the other with $\mathcal{D}$ and $\mathcal{E}$. The concentration states of $\mathcal{A}$, $\mathcal{B}$, $\mathcal{C}$, $\mathcal{D}$, $\mathcal{E}$ are represented as x_1, x_2, x_3, x_4, x_5 respectively. Input (I) is applied on $\mathcal{A}$ and the concentration of $\mathcal{E}$ is considered as output.

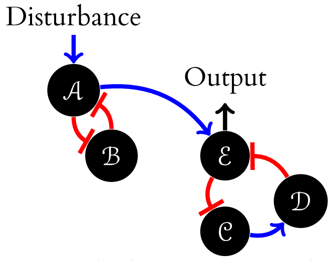

Fig S2. Proposed five node network which can not provide adaptation albeit satisfying the weaker condition for stability

From the network structure in S2, it can be seen that there are two loops involved in the network. One is engaging $\mathcal{A}$ and $\mathcal{B}$, another with $\mathcal{C}$, $\mathcal{D}$, $\mathcal{E}$ nodes. Both the feedback schemes are positive in nature. From the structure it can be intuitively seen that the network is controllable for any non-zero strength of the edge from $\mathcal{B}$ to $\mathcal{C}$. This can also be proved mathematically by evaluating the rank of the associated controllability matrix.
For this network to provide adaptation, the corresponding system matrix **A** after linearisation can be of the structure

$$\mathbf{A} = \begin{bmatrix} \alpha_{aa} & \alpha_{ab} & 0 & 0 & 0 \\ \alpha_{ba} & \alpha_{bb} & 0 & 0 & 0 \\ 0 & 0 & \alpha_{cc} & 0 & \alpha_{ce} \\ 0 & 0 & \alpha_{dc} & \alpha_{dd} & 0 \\ \alpha_{ea} & 0 & 0 & \alpha_{ed} & \alpha_{ee} \end{bmatrix} \quad (36)$$

Note, if there is no edge from $\mathcal{A}$ to $\mathcal{E}$, the network would be uncontrollable. The condition to be met for this five node network to provide adaptation is the following $|\tilde{\mathbf{A}}| = 0$, where $\tilde{\mathbf{A}} =$

$$\begin{bmatrix} \alpha_{ba} & \alpha_{bb} & 0 & 0 & 0 \\ 0 & 0 & \alpha_{cc} & 0 & \alpha_{ce} \\ 0 & 0 & \alpha_{dc} & \alpha_{dd} & 0 \\ \alpha_{ea} & 0 & 0 & \alpha_{ed} & \alpha_{ee} \end{bmatrix}$$ So, for $|\tilde{\mathbf{A}}|$ to be zero α_{cc} has to be zero. The next condition is concerning the stability of **A**. With $\alpha_{bb} = 0$ the determinant of **A** can be written as

$$|\mathbf{A}| = -\alpha_{ab}\alpha_{ba}\alpha_{bb}\alpha_{cc}\alpha_{ee} - \alpha_{ab}\alpha_{ba}\alpha_{ce}\alpha_{dc}\alpha_{ed} \tag{37}$$

Now, for the system to be Hurwitz stable, the determinant of **A** is necessarily of the sign $(-1)^5$=-1. This can be achieved in two ways 1) both the terms are negative or 2) Either one of them is negative with magnitude greater than that of the positive term. The first case leads to at least one negative feedback, preferably between $\mathcal{A}$ and $\mathcal{B}$. In the second case, if both the loops are of positive feedback and if

$$|\alpha_{ab}\alpha_{ba}\alpha_{bb}\alpha_{cc}\alpha_{ee}| < \alpha_{ab}\alpha_{ba}\alpha_{ce}\alpha_{dc}\alpha_{ed}$$

then the necessary condition for the Hurwitz stability of **A** is satisfied. But on a careful introspection, it can be seen that at least one of the eigenvalues of **A** is positive which goes to violate the Hurwitz stability condition for **A** thereby leading to instability. So, the above network structure can be ruled out.
This can be understood from the **A** matrix for these cases. To make the network controllable and able to provide adaptation, it is necessary to add an edge from the input to the output node. Although the addition of an element changes the spectrum of the overall matrix, the spectrum of the block matrices containing the loops other except one will not be changed. If all the loops are positive at least one of the eigenvalues of the block matrices will be positive leading to instability for the overall matrix. In the example of S2 the addition of an edge from $\mathcal{A}$ to $\mathcal{E}$ has changed the spectrum of **A** without changing the spectra of the block matrix $\begin{bmatrix} \alpha_{11} & \alpha_{12} \\ \alpha_{21} & \alpha_{22} \end{bmatrix}$. With $\mathcal{A}$ and $\mathcal{B}$ in positive feedback, one of the eigenvalues can be verified as positive, which leads to the violation of Hurwitz property of **A**.

4 Necessary information for simulation

In all of the cases except the Na-gated voltage channel, the network dynamics used for simulation purpose have been inspired from the Ma *et al* (2011) wherein a variant of Michaelis Meneten kinetics has been used for the computational study [3]. It has already been proven in subsection 3.1 of the supplementary text that for a negative feedback module to provide perfect adaptation at least one diagonal element in any of the columns (rows of **A**) concerning the loop has to be zero. For this to happen the dynamics of the node represented by that particular column (containing a zero diagonal) has to be independent of the concentration of that node itself. To this purpose, the values of Michaelis Menten constants have been chosen and the initial conditions are obtained by computing the initial steady state of the dynamical system.
The dynamics used for Voltage-gated Sodium channel has been inspired from the review paper on adaptation produced by James J. Ferell (2016). The dynamics is a variant of a simple mass-action kinetics along with the conservation of total concentration of the biochemical species [4].
We used the DEE toolbox in MATLAB simulink to simulate the models. DEE uses 'ode45s' as the default numerical differential equation solver. The necessary MATLAB and simulink files for the same can be found on https://github.com/RamanLab/SystemsTheoryAdaptation.

Two node network

Two node Enzymatic network; We adopted the Michaelis-Menten kinetics wherein the concentration of the biochemical species $\mathcal{A}$ and $\mathcal{B}$ in Fig S3 are considered as $x_1(t)$ and $x_2(t)$ respectively.

$$\dot{x_1} = 10^7 I \frac{1 - x_1}{10^6 + (1 - x_1)} - 12 \times 10^3 x_2 \frac{x_1}{10^3 + x_1} \tag{38}$$

$$\dot{x_2} = 8x_1 \frac{1 - x_2}{10^{-6} + (1 - x_1)} - 2\frac{x_2}{10^{-5} + x_2} \tag{39}$$

N/W Structure	A matrix (Given N node n/w $\mathbf{B} = [\beta \quad O_{N-1\times 1}]^T$)	Condition I	Condition II	Conclusion
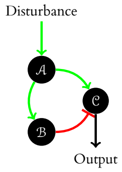	$\begin{bmatrix} a_{11}(-1) & 0 & 0 \\ a_{21}(+1) & a_{22}(-1) & 0 \\ a_{31}(+1) & a_{32}(-1) & a_{33}(-1) \end{bmatrix}$	$(\|\mathbb{S}\|_0 : \mathbb{S} := \{a_{31}, a_{21}a_{32}\}) \geqslant 1$	$a_{31}a_{22} - a_{21}a_{32} = 0$	✓
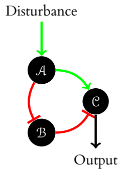	$\begin{bmatrix} a_{11}(-1) & 0 & 0 \\ a_{21}(-1) & a_{22}(-1) & 0 \\ a_{31}(+1) & a_{32}(-1) & a_{33}(-1) \end{bmatrix}$	$(\|\mathbb{S}\|_0 := \{a_{31}, a_{21}a_{32}\}) \geqslant 1$	$a_{31}a_{22} - a_{21}a_{32} = 0$	✗
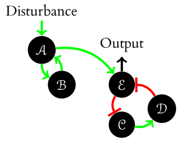	$\begin{bmatrix} a_{11}(-1) & a_{12}(+1) & 0 & 0 & 0 \\ a_{21}(+1) & a_{22}(-1) & 0 & 0 & 0 \\ 0 & 0 & a_{33}(-1) & 0 & a_{35}(-1) \\ 0 & 0 & a_{43}(+1) & a_{44}(-1) & 0 \\ a_{51} & 0 & 0 & a_{54}(-1) & a_{55}(-1) \end{bmatrix}$	$(\|\mathbb{S}\|_0 := \{a_{51}\}) \neq 0$	$a_{22} = 0$, $\mathrm{Re}(\mathrm{spec}(\mathbf{A})) < 0$	✗(Unstable)
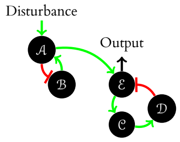	$\begin{bmatrix} a_{11}(-1) & a_{12}(-1) & 0 & 0 & 0 \\ a_{21}(+1) & a_{22}(-1) & 0 & 0 & 0 \\ 0 & 0 & a_{33}(-1) & 0 & a_{35}(+1) \\ 0 & 0 & a_{43}(+1) & a_{44}(-1) & 0 \\ a_{51}(+1) & 0 & 0 & a_{54}(-1) & a_{55}(-1) \end{bmatrix}$	$(\|\mathbb{S}\|_0 := \{a_{51}\}) \neq 0$	$a_{22} = 0$, $\mathrm{Re}(\mathrm{spec}(\mathbf{A})) < 0$	✓
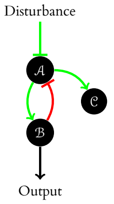	$\begin{bmatrix} a_{11}(-1) & a_{12}(+1) & 0 \\ a_{21}(-1) & a_{22}(-1) & 0 \\ a_{31}(+1) & 0 & a_{33}(-1) \end{bmatrix}$	$(\|\mathbb{S}\|_0 : \mathbb{S} := \{a_{31}\}) = 1$	$a_{22} = 0$,$\mathrm{Re}(\mathrm{spec}(\mathbf{A})) < 0$	✓

Table S1. Demonstration of the algorithm. $\|.\|_0 : \mathbb{S} \to \mathbb{R}$ refers to the number of non-zero elements in the set $\mathbb{S}$.

The initial conditions used for this simulation are obtained as $\begin{bmatrix} 0.25 \\ 0.75 \end{bmatrix}$

Voltage gated Na ion channel

As it can be seen in figure S4 the species $\mathcal{X}$ can stay in three possible states namely, active, inactive and off state. Since the total concentration is assumed to be constant, the underlying dynamical system boils down to a second order system. Here, we have considered $x_1(t)$ and $x_2(t)$ as the concentration of $\mathcal{X}_{\mathtt{off}}$ and $\mathcal{X}_{\mathtt{on}}$ respectively. As it can be seen that there exist a loss of controllability for the subsequent step chnges in the disturbance input.

$$\dot{x_1} = 1.8I(1 - x_1 - x_2) - x_1 \tag{40}$$

$$\dot{x_2} = x_1 \tag{41}$$

The corresponding initial condition is the origin as it is one of the steady states of the autonomous system.

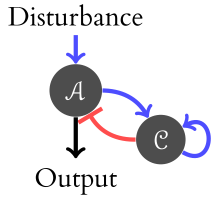

Fig S3. Two node network used for simulation

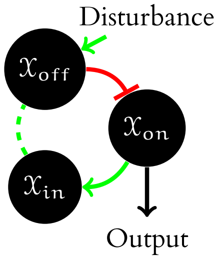

Fig S4. Voltage gated channels used for simulation

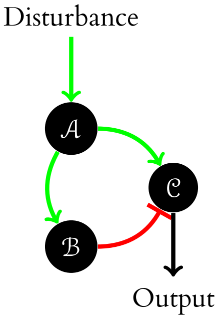

Fig S5. Architecture of a three-node IFFLP used for simulation

IFFLP

For the network in figure S5, the concentrations of $\mathcal{A}$, $\mathcal{B}$ and $\mathcal{C}$ are denoted as x_1, x_2 and x_3 respectively.

$$\dot{x_1} = 8I\frac{1 - x_1}{(0.0001 + (1 - x_1))} - 6x_1 \tag{42}$$

$$\dot{x_2} = 5x_1\frac{1 - x_2}{(0.00001 + (1 - x_2))} - 4x_2 \tag{43}$$

$$\dot{x_3} = 8x_1(1 - x_3)/(1.00001 - x_3) - 16x_2x_3 \tag{44}$$

The initial concentration vector are obtained as $\begin{bmatrix} 0.4 \\ 0.5 \\ 0.4 \end{bmatrix}$

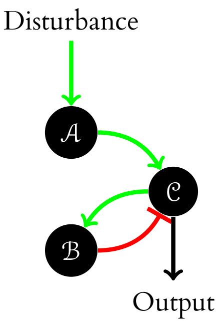

Fig S6. Architecture of a three-node NFBLB used for simulation

NFBLB

The corresponding dynamics used for the purpose of simulation is given by

$$\dot{x_1} = 8I\frac{1-x_1}{(10^{-6}+[1-x_1])} - 6x_1 \tag{45}$$

$$\dot{x_2} = 4x_3\frac{1-x_1}{(10^{-6}+[1-x_1])} - 2\frac{x_2}{(10^{-7}+x_2)} \tag{46}$$

$$\dot{x_3} = 16x_1(1-x_3) - 10x_3x_2 \tag{47}$$

The associated initial condition for the same is $\begin{bmatrix} 0.4 \\ 0.64 \\ 0.5 \end{bmatrix}$

IFFLP+NF

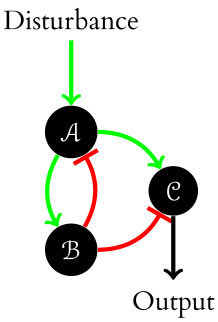

Fig S7. Architecture of a three-node IFFLP+NF used for simulation

$$\dot{x_1} = 8I\frac{1-x_1}{(0.0000001+(1-x_1))} - 14x_1x_2 \tag{48}$$

$$\dot{x_2} = 10x_1\frac{1-x_2}{(1.0000001-x_2)} - 4x_2 \tag{49}$$

$$\dot{x_3} = 8x_1\frac{(1-x_3)}{(1.000001-x_3)} - 16x_3x_2 \tag{50}$$

The corresponding initial condition used was $\begin{bmatrix} 0.2619 \\ 0.6547 \\ 0.2000 \end{bmatrix}$

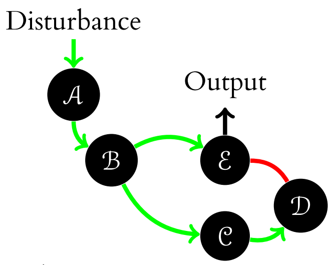

Fig S8. Five node IFFLP for simulation

Five node IFFLP

With the concentration of $\mathcal{A}$, $\mathcal{B}$, $\mathcal{C}$, $\mathcal{D}$, and $\mathcal{E}$ represented as x_1, x_2, x_3, x_4, and x_5 respectively, the underlying dynamics of the network is given by

$$\dot{x}_1 = 5I\frac{(1-x_1)}{(1.0000001-x_1)} - 8x_1 \tag{51}$$

$$\dot{x}_2 = 8x_1\frac{(1-x_2)}{(1.0000005-x_2)} - 6x_2 \tag{52}$$

$$\dot{x}_3 = 8x_2\frac{(1-x_3)}{(1.0000005-x_3)} - 4x_3 \tag{53}$$

$$\dot{x}_4 = 8x_3 - 5x_4 \tag{54}$$

$$\dot{x}_5 = 8x_2 - 8x_4x_5 \tag{55}$$

$$\tag{56}$$

The initial condition used for this simulation is $\begin{bmatrix} 0.1875 \\ 0.250 \\ 0.5000 \\ 0.8000 \\ 0.3125 \end{bmatrix}$

Five node NFBLB

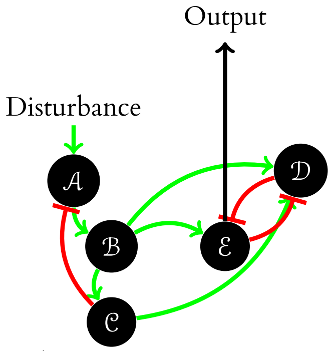

Fig S9. Five node IFFLP for simulation

The underlying dynamics can be written as

$$\dot{x}_1 = 4I(1 - x_1) - 32x_3x_1 \quad (57)$$
$$\dot{x}_2 = 10x_1(1 - x_2) - 2x_2 \quad (58)$$
$$\dot{x}_3 = 20x_2 - 6 \quad (59)$$
$$\dot{x}_4 = 3x_3 - 6x_5x_4 \quad (60)$$
$$\dot{x}_5 = 6x_2(1 - x_5) - 8x_4x_5 \quad (61)$$

The initial condition used for this simulation is $\begin{bmatrix} 0.0857 \\ 0.3000 \\ 0.4000 \\ 0.4500 \\ 0.3333 \end{bmatrix}$

IFFLP with a downstream

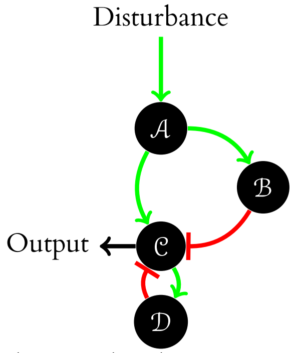

Fig S10. Architecture of IFFLP along with a downstream node

The corresponding network dynamics is given by

$$\dot{x}_1 = 5I - 6x_1 \quad (62)$$
$$\dot{x}_2 = 10x_1 - 4x_2 \quad (63)$$
$$\dot{x}_3 = 8x_1 - 3.2x_4x_3 - 6x_2 \quad (64)$$
$$\dot{x}_4 = 6x_3(1 - x_4) - 0.5 \quad (65)$$

The initial condition for the same can be written as $\begin{bmatrix} 0.25 \\ 0.625 \\ 0.0833 \\ 0 \end{bmatrix}$ It can be seen that although the network is able to perform perfect adaptation (for stability is not compromised with the connection to downstream system) the oscillatory output- concentration of $\mathcal{C}$ takes a negative value which does not carry a practical relevance. This definitely requires further detailed investigation.

NFBLB with downstream system

The concentration profiles of $\mathcal{A}$, $\mathcal{B}$, $\mathcal{C}$, and $\mathcal{D}$ are expressed as x_1, x_2, x_3, and x_4 respectively. The corresponding dynamical system can be written as

$$\dot{x}_1 = 8I - 6x_1 \quad (66)$$
$$\dot{x}_2 = 4x_3\frac{(1 - x_2)}{(1.0000004 - x_2)} - 2 \quad (67)$$
$$\dot{x}_3 = 16x_1(1 - x_3) - 25x_3x_2 - 2x_3x_4 \quad (68)$$
$$\dot{x}_4 = 8x_3(1 - x_4) - 3 \quad (69)$$

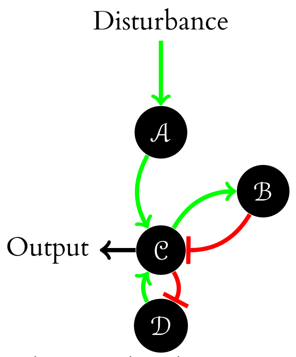

Fig S11. Architecture of NFBLB along with a downstream node

The associated initial condition is given by $\begin{bmatrix} 0.4000 \\ 0.2360 \\ 0.5000 \\ 0.2500 \end{bmatrix}$

References

1. Maybee J, Driessche P, Olesky D, Wiener G. Matrices, Digraphs, and Determinants. Society of Industrial and Applied Mathematics. 1989;10(4):500–519.
2. Robyn A, Lance L. The topological requirements for robust perfect adaptation in networks of any size. Nature Communications. 2018;9(13):1757–1769.
3. Ma W, Trusina A, El-Samad H, Lim A, Tang C. Defining Network Topologies that Can Achieve Biochemical Adaptation. Cell. 2009;138(4):760–773.
4. Ferell J. Perfect and Near-Perfect Adaptation in Cell Signaling. Cell Systems. 2016;2(7):62–67.
