# Supplementary material for: Discovering adaptation-capable biological network structures using control-theoretic approaches
Source: PLoS Comput Biol. 2022 Jan 21;18(1):e1009769. doi: 10.1371/journal.pcbi.1009769 (PMC8809615; doi:10.1371/journal.pcbi.1009769)
Supplement: S1 Table — (PDF) [file pcbi.1009769.s013.pdf]

N/W Structure	A matrix (Given N node n/w $\mathbf{B} = \begin{bmatrix}\beta & \mathbf{O}_{N-1\times 1}\end{bmatrix}^T$)	Condition I	Condition II	Conclusion
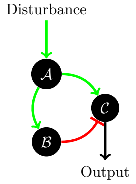	$\begin{bmatrix} a_{11}(-1) & 0 & 0 \\ a_{21}(+1) & a_{22}(-1) & 0 \\ a_{31}(+1) & a_{32}(-1) & a_{33}(-1) \end{bmatrix}$	$(\|\|\mathbb{S}\|\|_0 : \mathbb{S} := \{a_{31}, a_{21}a_{32}\}) \geq 1$	$a_{31}a_{22} - a_{21}a_{32} = 0$	✓
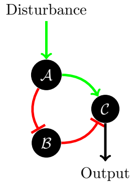	$\begin{bmatrix} a_{11}(-1) & 0 & 0 \\ a_{21}(-1) & a_{22}(-1) & 0 \\ a_{31}(+1) & a_{32}(-1) & a_{33}(-1) \end{bmatrix}$	$(\|\|\mathbb{S}\|\|_0 := \{a_{31}, a_{21}a_{32}\}) \geq 1$	$a_{31}a_{22} - a_{21}a_{32} = 0$	✗
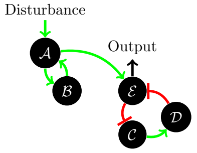	$\begin{bmatrix} a_{11}(-1) & a_{12}(+1) & 0 & 0 & 0 \\ a_{21}(+1) & a_{22}(-1) & 0 & 0 & 0 \\ 0 & 0 & a_{33}(-1) & 0 & a_{35}(-1) \\ 0 & 0 & a_{43}(+1) & a_{44}(-1) & 0 \\ a_{51} & 0 & 0 & a_{54}(-1) & a_{55}(-1) \end{bmatrix}$	$(\|\|\mathbb{S}\|\|_0 := \{a_{51}\}) \neq 0$	$a_{22} = 0,\ Re(spec(\mathbf{A})) < 0$	✗(Unstable)
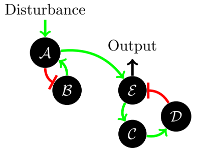	$\begin{bmatrix} a_{11}(-1) & a_{12}(-1) & 0 & 0 & 0 \\ a_{21}(+1) & a_{22}(-1) & 0 & 0 & 0 \\ 0 & 0 & a_{33}(-1) & 0 & a_{35}(+1) \\ 0 & 0 & a_{43}(+1) & a_{44}(-1) & 0 \\ a_{51}(+1) & 0 & 0 & a_{54}(-1) & a_{55}(-1) \end{bmatrix}$	$(\|\|\mathbb{S}\|\|_0 := \{a_{51}\}) \neq 0$	$a_{22} = 0,\ Re(spec(\mathbf{A})) < 0$	✓
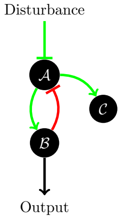	$\begin{bmatrix} a_{11}(-1) & a_{12}(+1) & 0 \\ a_{21}(-1) & a_{22}(-1) & 0 \\ a_{31}(+1) & 0 & a_{33}(-1) \end{bmatrix}$	$(\|\|\mathbb{S}\|\|_0 : \mathbb{S} := \{a_{31}\}) = 1$	$a_{22} = 0, Re(spec(\mathbf{A})) < 0$	✓

Table 1: Demonstration of the algorithm. $||.||_0 : \mathbb{S} \to \mathbb{R}$ refers to the number of non-zero elements in the set $\mathbb{S}$.
