# Supplementary material for: Discovering adaptation-capable biological network structures using control-theoretic approaches
Source: PLoS Comput Biol. 2022 Jan 21;18(1):e1009769. doi: 10.1371/journal.pcbi.1009769 (PMC8809615; doi:10.1371/journal.pcbi.1009769)
Supplement: Attachment — Submitted filename: Response to Reviewers.pdf [file pcbi.1009769.s014.pdf]

Response to the Reviewers

Priyan Bhattacharya, Karthik Raman, Arun K. Tangirala

November 16, 2021

Reviewer 1

1. *In this manuscript, the authors have studied the topic of discovering adaptation-capable biological networks. This has general interests in several research fields. The authors have simplified the problem to studying linear time-invariant systems. And they assume explicitly that one of the nodes be a controller for the adaptation system. Several conclusions interest me. For example, in section 2, the authors talked about how to get a non-zero response; and on page 13, it is neat to rewrite the determinant A into the addition of different components. But apart from these minor points, the current manuscript does not have enough progress to warrant a publication. The primary focus of the manuscript is to derive the adaptation network's topological features with control theory. But I feel that the approach is not fundamentally different than studying the Jacobian matrix of ODE equations. And the conclusions are largely the same as the previous findings.*

   Response: We thank the reviewer for pointing out the key contributions of the manuscript. While we partly agree with the argument that the Jacobian treatment of the problem is not new, we argue that the Jacobian treatment hitherto adopted has been limited in its capacity to characterise adaptation with respect to both precision and sensitivity. Therefore, in addition to the Jacobian analysis, we employed a number of well-known concepts inspired by control theory to deduce **precise mathematical (hence structural) conditions** both for non-zero sensitivity and infinite precision. This has enabled us to contribute to this field of study in the following ways.

   (A) The Jacobian treatment can be used in finding the conditions for infinite precision- an important quality for perfect adaptation but it does not guarantee non-zero sensitivity (which is also another important ask for adaptation). In this respect, our work demonstrates that the well-known concept of controllability is required over and above the Jacobian analysis.

   (B) The present work offers a systematic treatment in developing the conditions for perfect adaptation in presence of **step type** disturbance. Conversely, this also aids in a systematic understanding behind why certain networks such as Voltage gated Sodium channel can only perform perfect adaptation only once (fig. 3 of the revised manuscript).

   (C) A proof that the peak response time of any network admissible for perfect adaptation is minimum across a class of other network structures.

   (D) Although the negative feedback topology is touted to be capable of perfect adaptation, we, for the first time, showed that not all negative feedback motifs can do justice to this claim. Biochemical networks with negative feedback loops that contain an edge from the output to the input node can not provide perfect adaptation (refer to theorem 4 of the revised manuscript). In this sense, the necessary structural conditions for perfect adaptation put forth in this work are the strictest among the existing literature.

   (E) Most importantly, although negative feedback has been shown to be capable of perfect adaptation for networks of small size (not more than three nodes) for larger networks, this assertion has been largely on the basis of intuition (refer to page 53 of the supplementary section in the paper- *The topological requirements for robust perfect adaptation in networks of any size. Robyn A, Lance L. Nature Communications.2018;9(13):1757–1769.*). In this paper, we present a rigorous proof (refer to theorem 5 of the revised manuscript) of the fact that a balancer module of any size shall always be locally unstable if all the loops are positive. Therefore, it requires at least one negative feedback to produce perfect adaptation. A stronger condition for the stability of linearised system has enabled us to utilize the wealth of combinatorial matrix theory in order to accomplish this.

   Table 1 positions our contributions relative to the existing literature in the key aspects of methodology and results. As summarized in the table, the paper makes significant contributions in both the aspects, while making the same assumptions as as those in Araujo *et al.*.

   Finally, regarding the assumption of controller nodes, we take this opportunity to point out that (as shown in line 322 of the revised manuscript) the controller module can be conceived as the sub-network containing $N-2$ nodes of any $N-$node network. Evidently, the remaining two are the input and the output nodes. Therefore, for a three node network with different I/O nodes, there can be only one controller node possible.

2. *The authors have stated several conclusions in the manuscript. Based on the abstract, the authors have:*

   1. *The authors "translate the necessary qualitative conditions for adaptation to mathematical constraints using the language of systems theory, which we then map back as 'design requirements' for the underlying networks. We go on to prove that a protein network with different input–output nodes (proteins) needs to be at least of third-order in order to provide adaptation."*

   2. *The authors "show that the necessary design principles obtained for a three-node network in adaptation consist of negative feedback or a feed-forward realization. Interestingly, the design principles obtained by the proposed method remain the same for a network of arbitrary size and connectivity."*

3. *The authors "prove that the motifs discovered for adaptation are non-retroactive for a canonical downstream connection. This result explains how complex biological networks achieve robustness while keeping the core motifs unchanged in the context of a particular functionality."*

Points 1 and 2 are not new and have been demonstrated in different papers. The third part is not clear.

Response: We agree with the reviewer that a two-node network with different I/O nodes is known to be unable to meet the requirements of perfect adaptation. However, in this work, we, for the first time, using a systems theoretic approach have demonstrated that a two-node network is indeed capable of perfect adaptation provided the output is measured as the concentration of the same node that receives the disturbance input. This has been discussed at length in the section'two-node networks" of the supplementary text.

Secondly, the two-node and three-node networks serve as the building blocks of our approach that aims to discover the structural conditions on networks of any size for perfect adaptation. Araujo *et al.* (2018) in their seminal study, showed that feedback loops (balancer modules) or incoherent feedforward paths (opposer modules) is essential for perfect adaptation. Further, the insistence on the loops being negative was inspired from the demonstrations in small networks *i.e,* networks with at most five nodes.Therefore, negative feedback as the necessary element in the balancer module for perfect adaptation has been an extremely important conjectural assertion that required a rigorous proof which we presented in the current work.

Subsequently, we argue that the necessary structural requirements obtained by our approach are the strictest in the existing literature. We showed in theorem 4 of the revised manuscript that not all negative feedbacks with the characteristics of a balancer module can provide perfect adaptation. Negative feedback loops which do not contain any edge from the output to the input node can only be candidate units for adaptation.

Further, the third conclusion refers to lemma 6 of the revised manuscript. where we proved the modularity property of adaptation network in presence of a downstream system connected bidirectionally with the output node.

3. *The manuscript did not emphasize enough what's new in their study. What I find interesting is 1) the discussion on the network's response, which is not well-studied based on my knowledge. 2) to apply the method to higher-order networks. This topic has been studies but it is still very interesting if the authors could summarize new principles based on their own work. The authors have mentioned both parts, but the conclusions are not clear enough to guide further study for either theoretical or experimental biologists. And it's not clear from the writing whether the authors need to assume a single control node for large networks.*

   Response: The manuscript has been modified in accordance with this remark. We have revised the abstract section where we emphasised on the novel contributions of this work. Secondly, in the introduction section, we contextualized these contributions based on the existing literature. We hope that the revised version clearly highlights the contribution of this work. Finally, the revised version of the discussion section carries an effective road map for the future works to follow.

   As shown in line 322 of the revised manuscript, the controller module can be conceived as the sub-network containing $N-2$ nodes of any $N-$node network. Evidently, the remaining two are the input and the output nodes. Therefore, we do not assume a single control node.

4. *The writing of the manuscript needs to be improved. First, as mentioned before, the manuscript is not focused enough. Second, the use of figure space is poor. For example, figures 3, 4, and 5 look similar to what's shown in the figure, yet only after reading the figure legends I realized that they are trying to tell entirely different stories. And many times, I found the legends do not have a graphic counterpart in the figure. Third, some of the contents, like theorem 4, looks obvious to me. If there are no links between the two parts of the networks, it is hard to imagine how they will influence each other. Maybe the rigorousness of mathematical requires contents like this to be presented. I don't see the necessity from a practitioner's point of view.*

   Response: We have duly noted the suggestions by the reviewer and incorporated the appropriate changes in the original text. We have placed the theorem in the supplementary information in order to accommodate new additions in the revised manuscript.

Table 1: A comparative study between the current manuscript and the relevant literature

Reference	Contribution		Assumptions
	Methodological	Results	
Ma *et al* (2011)	• Adaptation characterised by Sensitivity and precision.<br>• Adopted computational screening approach for a three-enzyme network.<br>• Justified the result for a three-protein system using Jacobian analysis.	NFBLB or IFFLP are the only two three-node motifs admissible for adaptation.	• The network follows Michaelis-Menten kinetics.<br>• Each enzyme can have two states- active and inactive.
Tang *et al* (2016)	Composed a Jacobian-based approach to deduce the condition (the 'cofactor condition') for infinite precision.	All the existing admissible network structures in the literature obeys the proposed condition.	The elements of the Jacobian matrix do not change throughout the entire state space.
Araujo *et al* (2018)	• Utilizing the Jacobian as the digraph matrix, the admissible structures were obtained via the application of graph theory.<br>• Condition for infinite precision with respect to step input was obtained through Jacobian analysis.<br>• The sign of the determinant of the digraph matrix was used to obtain a week necessary condition for linear asymptotic stability.	• Balancer module (feedback loops with buffer node) or Opposer module (same as IFFLP) are the only two necessary modules for adaptation in networks of any size.<br>• Conjectured that balancer module should contain at least one negative feedback for stability.	The elements of the Jacobian matrix do not change their sign throughout the entire state space.

Wang *et al* (2020,21)	• Condition for infinite precision with respect to bounded input was obtained through Jacobian analysis.<br>• Utilizing the Jacobian as the digraph matrix, the admissible structures were obtained via the application of combinatorial matrix theory.	Feedback loops with buffer node / Haldane motifs or network motifs with opposing forward paths are the only two necessary modules for adaptation in networks of any size.	Similar to Araujo *et al.*
Current Manuscript	• Employed the notion of controllability to deduce the condition for non-zero sensitivity.<br>• Showed using control theory, that perfect adaptation ipso facto minimizes the peak time among the class of stable responses.<br>• Obtained the conditions for infinite precision for a network of any size with respect to staircase type disturbance.<br>• A set of N conditions concerning the sign of each coefficient in the characteristic polynomial of the digraph matrix was derived for the local asymptotic stability of the system. This serves as the strongest necessary conditions for stability among the ones in the literature.	• Proved Araujo *et al's* conjecture.<br>• Negative feedback that contains the edge from output to the input node cannot provide adaptation.<br>• The adaptive response remains unchanged in the presence of a downstream system.	Similar to Araujo *et al.*

Reviewer 2

1. *The manuscript is clearly-written and describes a timely subject that is of interest in the field of understanding and designing biological feedback mechanisms.*

   However, I do have a major concern: I am not clear on how the work distinguishes itself from several previously-published papers. I hope that the authors can address the ways in which the current work differentiates itself from the key results reported in the following:

   Ref 7 - Wang et al.. (2016) - This is referenced in passing in the Introduction, as a member of the group of graph-theory approaches to the problem, but it seems that the results in that paper have considerable overlap with the ones discussed in the Results section, including the nature of topological constraints on networks with various numbers of nodes.

   Response: The authors thank the reviewer for the constructive suggestions. While we agree with the reviewer that the topologies discovered by Wang *et al* in two of their subsequent works (*Golubitsky M, Wang Y. Infinitesimal homeostasis in three-node input–output networks. Journal of Mathematical Biology. 2020;80(4):1163–1185.doi:10.1007/s00285-019-01457-x* and *Wang Y, Huang Z, Antoneli F, Golubitsky M. The structure of infinitesimal homeostasis in input–output networks. Journal of MathematicalBiology. 2021;82(7):1–43*) are similar to the current text we claim that the results consequent of the proposed methodology are different from Wang *et al.*(2021) in three accounts.

   (A) Unlike Wang *et al.*, we applied the well-known concept of controllability in the derivation of the admissible motifs for perfect adaptation. In fact, connecting the requirement of non-zero sensitivity to the condition of controllability enabled us to understand and explain the response of a class of networks such as voltage-dependent sodium channels that can provide adaptation only once (*i. e.* for the first step change) when subjected to a staircase-type disturbance.

   (B) We argue in theorem 1 of the revised manuscript the structural requirements of perfect adaptation also minimizes the peak time of the response.

   (C) Most importantly, we claim that the necessary structural requirements obtained by our approach are the strictest in the existing literature. We substantiate our claim on the basis of two new results. i) Wang *et al.* (2021) proved that negative feedback loop provides exponential stability for a three node network but it still remains unclear what happens for larger networks. We address this question by rigorously proving that in the case of any network with no incoherent feedforward characteristics, it can only provide adaptation if it contains at least one negative feedback and ii) secondly, not all negative feedbacks can provide perfect adaptation. Only those which do not contain any edge from the output to input node can be candidate motifs for adaptation.

   A brief discussion on the works of Wang *et al.* (2021) has been presented in the introduction section of the revised manuscript (from line no. 89-98).

2. *Ref 11 - Ma et al.. (2011) - This is discussed extensively in the current manuscript, but I'm hoping the authors can address the analysis Ma et al.. included in their Supplementary information section. In that section, a linearized form of the dynamics of three-node networks is analyzed using matrix approaches, similarly to the way it has been done in the current manuscript.*

   Response: Although the authors agree with the reviewer that the analysis performed by Ma *et al.* (2016) on a three node network with different I/O nodes in the supplementary text can act as an appropriate starting point it is to be noted that the present study deals with the most generic situation of networks of any size and configuration. Furthermore, in this context, the current work uses a stronger set of conditions for stability and discovers new conditions for non zero sensitivity (controllability at each steady-state) in order to find out network structures of any size that can attain perfect adaptation.

   A short and comprehensive discussion on the same has been added in the revised manuscript with line numbers ranging from 51-55.

3. *Ref 34 - Robyn P. Araujo and Lance A. Liotta (2018). [And Araujo and Liotta cite, in their Methods, a previous publication by Tang and McMillen (2016), which they describe as an alternative but mathemtically equivalent version of their approach - Zhe F. Tang and David R. McMillen (2016), Design principles for the analysis and construction of robustly homeostatic biological networks. Journal of Theoretical Biology. http://dx.doi.org/10.1016/j.jtbi.2016.06.036]*

   Ref 34 is addressed briefly in the discussion, but since both of the above papers address the constraints on networks that aim at perfect adaptation/homeostatic disturbance rejection, it would be helpful to have a more detailed discussion of the differences between the current work and the previous reports.

   Response: While we partly agree with the argument that the Jacobian treatment of the problem is not new, we argue that the Jacobian treatment hitherto adopted has been limited in its capacity to characterise adaptation with respect to both precision and sensitivity. Therefore, in addition to the Jacobian analysis, we employed a number of well-known concepts inspired by control theory to deduce **precise mathematical (hence structural) conditions** both for non-zero sensitivity and infinite precision. This has enabled us to contribute to this field of study in the following ways.

   (A) The Jacobian treatment can be used in finding the conditions for infinite precision- an important quality for perfect adaptation but it does not guarantee non-zero sensitivity (which is also another important ask for adaptation). In this respect, our work demonstrates that the well-known concept of controllability is required over and above the Jacobian analysis.

Table 1: A comparative study between the current manuscript and the relevant literature

Reference	Contribution		Assumptions
	Methodological	Results	
Ma *et al* (2011)	• Adaptation characterised by Sensitivity and precision.<br>• Adopted computational screening approach for a three-enzyme network.<br>• Justified the result for a three-protein system using Jacobian analysis.	NFBLB or IFFLP are the only two three-node motifs admissible for adaptation.	• The network follows Michaelis-Menten kinetics.<br>• Each enzyme can have two states-active and inactive.
Tang *et al* (2016)	Composed a Jacobian-based approach to deduce the condition (the 'cofactor condition') for infinite precision.	All the existing admissible network structures in the literature obeys the proposed condition.	The elements of the Jacobian matrix do not change throughout the entire state space.
Araujo *et al* (2018)	• Utilizing the Jacobian as the digraph matrix, the admissible structures were obtained via the application of graph theory.<br>• Condition for infinite precision with respect to step input was obtained through Jacobian analysis.<br>• The sign of the determinant of the digraph matrix was used to obtain a week necessary condition for linear asymptotic stability.	• Balancer module (feedback loops with buffer node) or Opposer module (same as IFFLP) are the only two necessary modules for adaptation in networks of any size.<br>• Conjectured that balancer module should contain at least one negative feedback for stability.	The elements of the Jacobian matrix do not change their sign throughout the entire state space.

Wang *et al* (2020,21)	• Condition for infinite precision with respect to bounded input was obtained through Jacobian analysis.<br>• Utilizing the Jacobian as the digraph matrix, the admissible structures were obtained via the application of combinatorial matrix theory.	Feedback loops with buffer node / Haldane motifs or network motifs with opposing forward paths are the only two necessary modules for adaptation in networks of any size.	Similar to Araujo *et al.*
Current Manuscript	• Employed the notion of controllability to deduce the condition for non-zero sensitivity.<br>• Showed using control theory, that perfect adaptation ipso facto minimizes the peak time among the class of stable responses.<br>• Obtained the conditions for infinite precision for a network of any size with respect to staircase type disturbance.<br>• A set of N conditions concerning the sign of each coefficient in the characteristic polynomial of the digraph matrix was derived for the local asymptotic stability of the system. This serves as the strongest necessary conditions for stability among the ones in the literature.	• Proved Araujo *et al's* conjecture.<br>• Negative feedback that contains the edge from output to the input node cannot provide adaptation.<br>• The adaptive response remains unchanged in the presence of a downstream system.	Similar to Araujo *et al.*

(B) The present work offers a systematic treatment in developing the conditions for perfect adaptation in presence of **step type** disturbance. Conversely, this also aids in a systematic understanding behind why certain networks such as Voltage gated Sodium channel can only perform perfect adaptation only once (fig. 3 of the revised manuscript).

(C) A proof that the peak response time of any network admissible for perfect adaptation is minimum across a class of other network structures.

(D) Although the negative feedback topology is touted to be capable of perfect adaptation, we, for the first time, showed that not all negative feedback motifs can do justice to this claim. Biochemical networks with negative feedback loops that contain an edge from the output to the input node can not provide perfect adaptation (refer to theorem 4 of the revised manuscript). In this sense, the necessary structural conditions for perfect adaptation put forth in this work are the strictest among the existing literature.

(E) Most importantly, although negative feedback has been shown to be capable of perfect adaptation for networks of small size (not more than three nodes) for larger networks, this assertion has been largely on the basis of intuition (refer to page 53 of the supplementary section in the paper- *The topological requirements for robust perfect adaptation in networks of any size. Robyn A, Lance L. Nature Communications.2018;9(13):1757–1769.*). In this paper, we present a rigorous proof (refer to theorem 5 of the revised manuscript) of the fact that a balancer module of any size shall always be locally unstable if all the loops are positive. Therefore, it requires at least one negative feedback to produce perfect adaptation. A stronger condition for the stability of linearised system has enabled us to utilize the wealth of combinatorial matrix theory in order to accomplish this.

The authors seek reviewer's attention to line numbers ranging from 70-98 of the revised manuscript that summarizes the work done by Tang and McMillen (2016), Araujo *et al* (2018), and Wang *et al* (2021). Furthermore, line numbers from 99-106 and 112-119 also discuss the major contributions of this work with respect to the work done by Araujo *e al*. Table 1 positions our contributions relative to the existing literature in the key aspects of methodology and results. As summarized in the table, the paper makes significant contributions in both the aspects, while making the same assumptions as as those in Araujo *et al.*.

Reviewer 3

First of all, I would like to sincerely apology to the editors and authors for the delay in my review. I consider that the article is of interest to the computational biology community. However, the following comments need to be addressed before being accepted for publication.
Major comments:

1. *Type of networks under study. There is a major confusion in the article concerning the type of networks under study. Ma et al., did the study for enzymatic networks (the states of the ODE system are enzyme concentrations). Here, the authors refer all the time to enzymatic networks, but when what they define the system's under study (starting in line 144), it seems they are talking about GRNs: gene regulatory networks (also denoted as transcriptional networks). Note that the type of kinetics of GRNs (Hill or others), differs from the kinetics of enzymatic networks (Mass action or Michaelis Menten typically). Also, it does not make sense in general to talk about mass conservation in typical GRNs. The authors need to clarify what type of networks they are dealing with, use the right kinetics, be coherent through the text, and discuss the results with respect to other studies taking this into account. (In the literature, both GRNs and enzymatic networks have been studied in terms of adaptation). The methodology presented by the authors is independent on the kinetics, and therefore applicable to GRNs, enzymatic, and any other type of biological networks (see comment below).*

   Ma et al., in their work, study enzymatic networks (using a rather particular kinetics), and they suggest in the text that the conclusions for networks involving transcription might differ from those for enzymatic networks. However, the linearization strategy proposed by the authors of this work, justified by the Hartman-Grobman theorem, seems to imply that kinetics are not relevant and the conclusions valid for enzymatic and transcriptional networks. This dichotomy deserves further discussion in the text. Also, if it is valid for general kinetics, it would be better to talk through the text about 'biochemical networks or "biological networks" instead of focusing on a particular class (enzymatic, grn)...

   Response: The authors are thankful to the reviewer for a comprehensive review. The suggestions have been incorporated in the revised manuscript. As pointed out by the reviewer, we have replaced the enzymatic networks with biochemical networks.

   The conclusions obtained by the analysis in linear domain as performed in this work remains valid as long as the elements in the Jacobian matrix do not change its sign through out the state space. It can be seen that for biologically acceptable parameter space and concentration profiles, this condition is satisfied by most of the reaction kinetics used in the literature.

2. *Mass conservation. Mass conservation seems to be used through the text without a clear justification, and introduces ambiguity regarding "the order of the system".*

   Without mass conservation, as it is the case always in GRNs, N is the number of nodes, the number of states of system, and the number of ODEs. In this case, the meaning of the order of the system=N, is clear. In line 153, it is stated that "In passing, it may be noted that in the presence of any algebraic constraints on the states (e.g. due to conservation laws), an N-node network corresponds to a reduced order dynamical system" this introduces confusion and might be removed. The order of the system, the number of nodes of a network, and the meaning of N needs to be clarified in the text. If N is used in the text to the number of Nodes, and the number of nodes are the proteins involved, then the number of state variables of the system is On the other hand, only in cases where X_1, X_2 and X_3 represent different states of the same protein modified through phosphorylation, methylation etc we can have the mass conservation law $X_1+X_2+X_3$ = constant, otherwise (in case of GRNs for example) it does not make sense. The presence of mass conservation must be "physically" justified through the text.

   Response: The proposed corrections are duly noted and the text is modified accordingly. The use of mass conservations have only been used for voltage-gated Na^+ channels.

3. *Context, originality and generality*

   (i) *The methodology presented (Figure 2) relies on the linearization of the system and then exploiting the theory of linear systems and control theoretic associated concepts (controllability, etc). The authors apply the methodology to study conditions for adaptation.*

   The authors justify the use of the linearization using Hartman-Grobman, which they claim it applies in the case of adaptation because, in fact the initial and final states of the process are stable steady states. However, this is not the case for many other functionalities of interest in systems biology, and then the methodology wouldn't be that general. The conditions under which the methodology applies must then be clearly stated and mentioned from the beginning.

   Response: The authors agree that the analysis in the linearised domain is not possible for every other functionality.

   (ii) *Other previous studies of adaptation by different approaches (exhaustive exploration, optimization. . .) do not rely on linearizations. Has linearization been used previously to study adaptation (and/or other motif-functionalities)? A bit of contextualization is missing in this regard, which could clarify better the originality and contribution of the paper. If this is the first it should be somehow emphasized.*

   Response: Almost all of the control-theoretic approaches on adaptation relied on the analysis of the linearised system. Drenstig *et al.* (Drengstig T, Ueda H, Ruoff P. Predicting perfect adaptation motifs in reaction kinetic networks. J Phys Chem B. 2008;112(15):16752–16758. doi:10.1021/jp806818c) and Allgower *et al.* (Waldherr S, Streif S, Allgöwer F.

Design of biomolecular network modifications to achieve adaptation. IET Syst Biol. 2012;6(14):223–31. doi:10.1049/iet-syb.2011.0058) deduced the systems-theoretic conditions for adaptation using a linearized model. Later Bhattacharya *et al.* (Bhattacharya P, Raman K, Tangirala A. A systems-theoretic approach towards designing biological networks for perfect adaptation. IFACPapersOnline.2018;51(5):307–312. doi:10.1016/j.ifacol.2018.05.033.) used a linear time-invariant systems-theoretic framework to deduce the structural requirements for adaptation for a three node network. Araujo *et al.* (The topological requirements for robust perfect adaptation in networks of any size.Robyn A, Lance L. Nature Communications.2018;9(13):1757–1769) and Wang *et al.* (Wang Y, Huang Z, Antoneli F, Golubitsky M. The structure of infinitesimal homeostasis in input–output networks. Journal of Mathematical Biology. 2021;82(7):1–43) also used Jacobian analysis to obtain the same for networks of any size.

In the current work, we used a larger set of concepts pertaining to linear systems theory i) to prove that the structural requirements of adaptation ipso facto minimize the peak time of the response, ii) to explain the phenomena of certain networks (such as voltage-dependent Sodium gate channels) providing adaptation only for limited accounts, and most importantly, iii) to find out the most strict set of necessary structural conditions (a strict subset of the previously obtained structural requirements) for any network to provide adaptation.

A concise presentation of the contribution of this work in the light of the existing literature has been presented in Table 1.

4. *Perfect adaptation is less general than adaptation. The study refers to perfect adaptation and this should be clarified already at the introduction. Also, it is said through the text (line 36) that Ma et al. study perfect adaptation but their study deals with adaptation in general.*

   Response: The authors agree with the aforementioned observation of the reviewer that perfect adaptation is indeed a specific case of adaptation. We have declared the theme of the work in line no. 101 of the revised manuscript. Further, line numbers 155-156 of the revised manuscript clarify the scope of this work in this regard.

5. *Reproducibility. Please provide the full information needed for simulation (not only the equations, also the initial conditions etc) such that the reader can easily reproduce results. It is highly recommended to provide scripts for simulations.*

   Response: The informations on the initial conditions for each of the simulation have been provided in the SI. The necessary scripts and simulink models are available on `https://github.com/RamanLab/SystemsTheoryAdaptation`.

6. *Models. Where the models in the SI come from? and the particular values of the parameters used? please provide references/context for the models used.*

   Response: The models used in the SI are inspired from Michaelis-Menten kinetics of an enzymatic network where each protein can be either in active and inactive state. Further, the rationale behind each model has been included in the SI.

Minor Comments:

1. *Please revise citations along the text, some of them are not correct. Please use the correct and standard form to cite the papers using the first author's name. For example, use Ma et al.. (2006) instead of Tang et al. (2006). In the same way, it is not "Banga et al. (2016) conceived" It is Otero-Muras and Banga (please do not invisibilize the author woman who actually conceived and performed that work). By the way, we further used the MINLP multiobjective optimization methodology to study (robust) adaptation (Otero-Muras and Banga 2019, Processes) in line with previous works by Barnes et al. 2011 (PNAS, 108) and Lormeau et al. 2017 (IFAC Papers online 50) that also tackled the problem of robust adaptation by different methods.*

   Response: We have revised the citation pattern through out the entire text. The authors are sincerely sorry for certain unintentional flaws. Further, the suggested resources have also been added in the list of citations.

2. *Typos. There are several typos along the text (see for example line 620-621). Also, the punctuation marks after the equations are in many cases incorrectly located at the beginning of the next text line.*

   Response: The authors have scanned through the text and revised the typographical errors. The authors are extremely grateful for the minute attention of the reviewer.

Table 1: A comparative study between the current manuscript and the relevant literature

Reference	Contribution		Assumptions
	**Methodological**	**Results**	
Ma *et al* (2011)	• Adaptation characterised by Sensitivity and precision.<br>• Adopted computational screening approach for a three-enzyme network.<br>• Justified the result for a three-protein system using Jacobian analysis.	NFBLB or IFFLP are the only two three-node motifs admissible for adaptation.	• The network follows Michaelis-Menten kinetics.<br>• Each enzyme can have two states- active and inactive.
Tang *et al* (2016)	Composed a Jacobian-based approach to deduce the condition (the 'cofactor condition') for infinite precision.	All the existing admissible network structures in the literature obeys the proposed condition.	The elements of the Jacobian matrix do not change throughout the entire state space.
Araujo *et al* (2018)	• Utilizing the Jacobian as the digraph matrix, the admissible structures were obtained via the application of graph theory.<br>• Condition for infinite precision with respect to step input was obtained through Jacobian analysis.<br>• The sign of the determinant of the digraph matrix was used to obtain a week necessary condition for linear asymptotic stability.	• Balancer module (feedback loops with buffer node) or Opposer module (same as IFFLP) are the only two necessary modules for adaptation in networks of any size.<br>• Conjectured that balancer module should contain at least one negative feedback for stability.	The elements of the Jacobian matrix do not change their sign throughout the entire state space.

Wang *et al* (2020,21)	• Condition for infinite precision with respect to bounded input was obtained through Jacobian analysis.<br><br>• Utilizing the Jacobian as the digraph matrix, the admissible structures were obtained via the application of combinatorial matrix theory.	Feedback loops with buffer node / Haldane motifs or network motifs with opposing forward paths are the only two necessary modules for adaptation in networks of any size.	Similar to Araujo *et al.*
Current Manuscript	• Employed the notion of controllability to deduce the condition for non-zero sensitivity.<br><br>• Showed using control theory, that perfect adaptation ipso facto minimizes the peak time among the class of stable responses.<br><br>• Obtained the conditions for infinite precision for a network of any size with respect to staircase type disturbance.<br><br>• A set of N conditions concerning the sign of each coefficient in the characteristic polynomial of the digraph matrix was derived for the local asymptotic stability of the system. This serves as the strongest necessary conditions for stability among the ones in the literature.	• Proved Araujo *et al's* conjecture.<br><br>• Negative feedback that contains the edge from output to the input node cannot provide adaptation.<br><br>• The adaptive response remains unchanged in the presence of a downstream system.	Similar to Araujo *et al.*
